# Association of *CYP2E1* gene polymorphisms with bladder cancer risk: A systematic review and meta-analysis

**DOI:** 10.1097/MD.0000000000011910

**Published:** 2018-09-28

**Authors:** Xiangrui Yin, Wei Xiong, You Wang, Wei Tang, Wang Xi, Shengqiang Qian, Yu Guo

**Affiliations:** a Department of Urology, Chongqing Hospital of Traditional Chinese Medicine; b Department of Urology, First Affiliated Hospital, Medical University of Chongqing, Chongqing, China.

**Keywords:** bladder cancer, *CYP2E1* gene polymorphisms, human cytochrome P450, meta-analysis, systematic review

## Abstract

**Background:**

Human cytochrome P450 (CYP) is an enzyme responsible for the metabolic activation of many carcinogens, including nitrosamines. *CYP2E1* represents a major CYP isoform and is expressed in the human urothelial cells. Recent studies have investigated the association of *CYP2E1* gene polymorphisms with bladder cancer risk but have shown contradictory results. Hence, we performed a systematic literature review and meta-analysis to assess the association between *CYP2E1* gene polymorphisms and bladder cancer.

**Methods:**

Systematic literature searches were conducted with PubMed, Excerpt Medica Database, Science Direct/Elsevier, China National Knowledge Infrastructure, and the Cochrane Library up to January 2018 for studies that involved the association of *CYP2E1* gene polymorphisms with bladder cancer risk. A meta-analysis was performed with Review Manager and Stata software. Combined odds ratios (ORs) were identified with 95% confidence intervals (CIs) in a random or fixed effects model.

**Ethics:**

The protocol was approved by the institutional review board of each study center. Written informed consent will be obtained from all patients before registration, in accordance with the Declaration of Helsinki.

**Results:**

Eight studies were identified, including 1733 cases of bladder cancer and 1814 normal controls. Our results illustrated that there are significant associations between *CYP2E1* gene polymorphisms and bladder cancer in all genetic models (*P* < .05). The combined ORs and 95% CIs were as follows for each model: additive model [OR 0.56; 95% CI (0.38–0.82)]; dominant model [OR 0.79; 95% CI (0.67–0.93)]; recessive model [OR 0.61; 95% CI (0.41–0.89)]; codominant model [OR 0.80; 95% CI (0.67–0.96)]; allele model [OR 0.75; 95% CI (0.59–0.95)]. A subgroup study showed that there are also significant associations between *CYP2E1* gene polymorphisms and bladder cancer in Asian people. However, there are no significant associations between *CYP2E1* gene polymorphisms and bladder cancer in Caucasian populations.

**Conclusions:**

The present study provides evidence for an association between *CYP2E1* gene polymorphisms and bladder cancer progression, and suggests that *CYP2E1* gene polymorphisms might be a protective factor against bladder cancer in Asian people. However, studies with larger sample sizes are needed to confirm the correlation between *CYP2E1* gene polymorphisms and bladder cancer.

## 1 Introduction

Globally, bladder cancer is the 10th most common cancer and accounts for 3.3% of all malignancies,^[1,2]^ with the highest incidence rates reported in Europe, North America, and Australia.^[3,4]^ Its incidence and mortality is the highest among urinary-system tumors, and the majority (approximately 70%) of cases occurs in men.^[5]^ The risks of bladder cancer are associated with tobacco smoking, some industrially related carcinogenic compounds amines, and amides, and some anticancer drugs such as phosphoramide mustards.^[6,7]^ However, not all people exposed to these risk factors are develops bladder cancer, suggests that the variation in individual susceptibility may play an important role to bladder carcinogenesis.

Currently, many studies have reported that genetic factors may play an important role in the risk of developing bladder cancer; many ultimate carcinogens and their genotoxicity functions require enzymatic bioactivation.^[10,11]^ Therefore, phase I and phase II metabolizing enzymes genes may be an important risk factors of developing cancer. Human cytochrome P450 (CYP) is a phase I enzyme play an important role for the metabolic activation of many procarcinogens.^[12]^*CYP2E1* represents a major CYP isoform, and in the liver and to lesser extent in other organs and tissues, including human urothelial cells are constitutively expressed this gene.^[13]^ Many low-molecular-weight carcinogens, such as vinyl chloride, benzene, and tobacco-specific nitrosamines require this critical enzyme to metabolic activation.^[14,15]^

However, although recent studies have investigated the association of *CYP2E1* gene polymorphisms with bladder cancer risk, the reported results were inconsistent, with some studies reporting that *CYP2E1* gene polymorphisms may increase the risk of bladder cancer, but not others.^[16–23]^ Therefore, we systematically reviewed the available literature and performed a meta-analysis to evaluate the association of *CYP2E1* gene polymorphisms with bladder cancer risk, which might shed valuable insights on our understanding of the biology of bladder cancer.

## 2 Methods

### 2.1 Literature search

This meta-analysis was restricted to published studies that investigated *CYP2E1* gene polymorphisms and bladder cancer risk. Two independent reviewers searched PubMed, Excerpt Medica Database, Science Direct/Elsevier, MEDLINE, China National Knowledge Infrastructure, and the Cochrane Library from inception to January 2018; the language or study type was not restricted. The search terms combined text words and MeSH terms. For example, the search terms for *CYP2E1* gene were “cytochrome P450 family” or “cytochrome P450 2E1” or “cyp2E1,” “cyp2e1” or “CYP2E1”; terms for bladder cancer included “bladder cancer” or “bladder neoplasms” or “cancer of bladder” or “neoplasms, bladder”; search terms for polymorphism included “SNP” or “single nucleotide polymorphism” or “polymorphism” or “variation” or “mutation.” All related articles and abstracts were retrieved. In addition, references cited within relevant reviews were retrieved by hand, and only full articles were searched.

### 2.2 Eligibility criteria

#### 2.2.1 Inclusion criteria

Included studies tested the association of *CYP2E1* gene polymorphisms with bladder cancer. The case groups were patients with bladder cancer and the controls were normal people. Genotyping for SNPs of *CYP2E1* was conducted using polymerase chain reaction restriction fragment length polymorphism. Available data were extracted from the article, including eligible and genotyped cases and controls, and number of cases and controls for each *CYP2E1* genotype.

#### 2.2.2 Exclusion criteria

Studies were excluded if they were case reports, meeting reports or review articles, published only in abstract form, included no control population, reported no available genotype frequency, or were a duplication of previous publications.

### 2.3 Study selection and validity assessment

Two independent reviewers screened the titles and abstracts of all citations from the literature search. All relevant studies that appeared to meet the eligibility criteria were retrieved. If an ambiguous decision was made based on the title and abstract, full texts were needed for the analysis. The final decision of eligible studies was made by reviewing the articles. Disagreements were resolved by consensus or a third reviewer.

### 2.4 Data extraction and statistical analysis

Data included demographic data (authors, year of publication, country, number, genotyping methods) and outcome data of eligible and genotyped cases and controls, plus number of cases and controls for each *CYP2E1* genotype. Three reviewers extracted data from the studies. Disagreements were resolved by consensus. A quantitative meta-analysis was performed by 2 reviewers using Review Manager software (version 5.2, The Nordic Cochrane Centre, The Cochrane Collaboration, 2012, Copenhagen, Denmark) and Stata software (version 12.0, College Station, TX). Available data were analyzed in the meta-analysis.

To calculate combined odds ratio (OR) and 95% confidence intervals (CIs), heterogeneity was assessed by the *P* value and the *I* square statistic (*I*^2^) in the pooled analyses, which represented the percentage of total variation across studies. If the *P* value was <.1 or the *I*^2^ value was >50%, the summary estimate was analyzed in a random-effects model. Otherwise, a fixed-effects model was applied. We investigated the association between *CYP2E1* gene polymorphisms and bladder cancer risk in allelic [allele (C2 vs C1)], additive (C2C2 vs C1C1), dominant (C2C2 and C2C1 vs C1C1), recessive (C2C2 vs C2C1 and C1C1), and codominant models (C2C1 vs C1C1). In addition, publication biases were detected by visual symmetry of funnel plots, with asymmetry suggesting possible publication bias. If the *P*-value was less than 0.05, publication bias was determined to exist.

## 3 Results

### 3.1 Characteristics of the included studies

**Figure 1:**
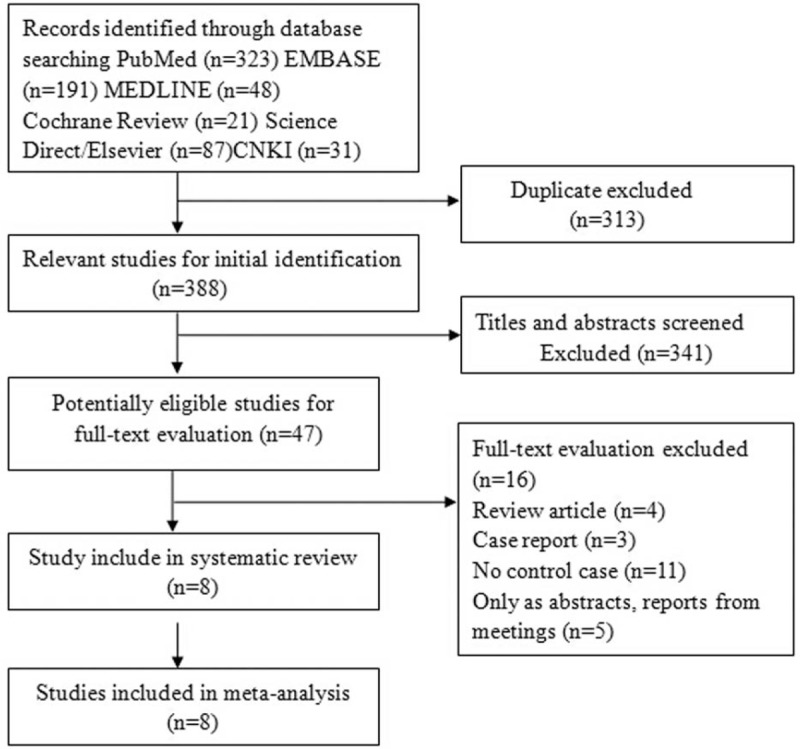
Flow diagram of the selection of eligible studies. CNKI = China National Knowledge Infrastructure, EMBASE = Excerpt Medica Database.

Figure 1 shows a detailed review process. A total of 701 unduplicated studies were identified, 8 studies were ultimately selected according to eligibility criteria, and all reviewers were in agreement regarding the inclusion of all 6 articles.

Table 1 summarizes general data from the 6 studies. All retrieved studies involved 1733 patients with bladder cancer and 1814 normal controls. All of these studies reported exclusion/inclusion criteria,^[16–23]^ and all tested for *CYP2E1* gene polymorphisms using restriction fragment-length-polymorphism analysis after polymerase chain reaction amplification.

**Table 1:**
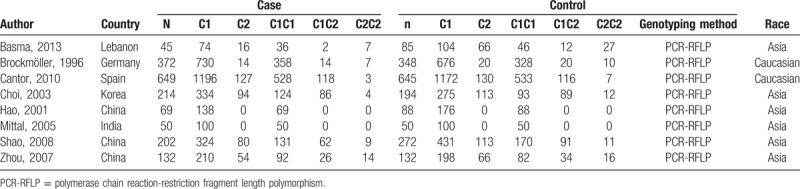
Characteristics of the included studies.

### 3.2 Meta-analysis

**Figure 2:**
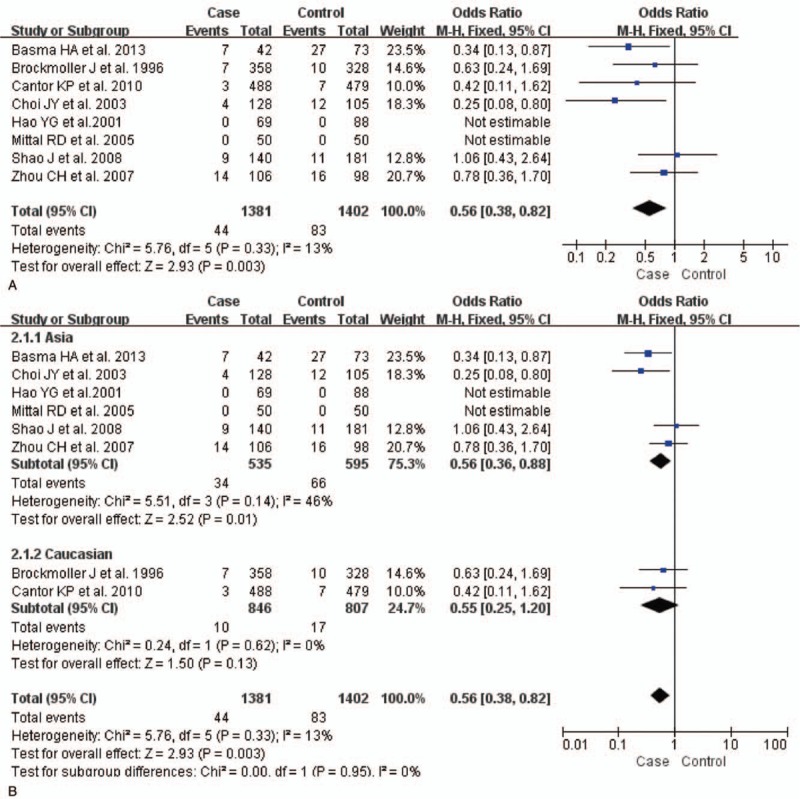
Forest plot showing the meta-analysis outcomes of the additive model. CI = confidence interval.

**Figure 3:**
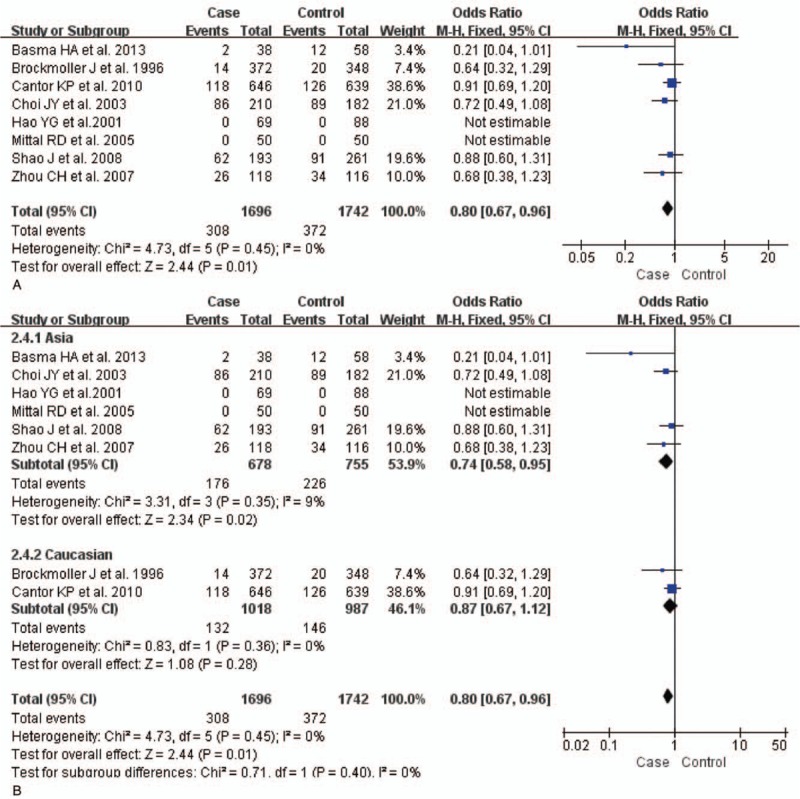
Forest plot showing the meta-analysis outcomes of the codominant model. CI = confidence interval.

**Figure 4:**
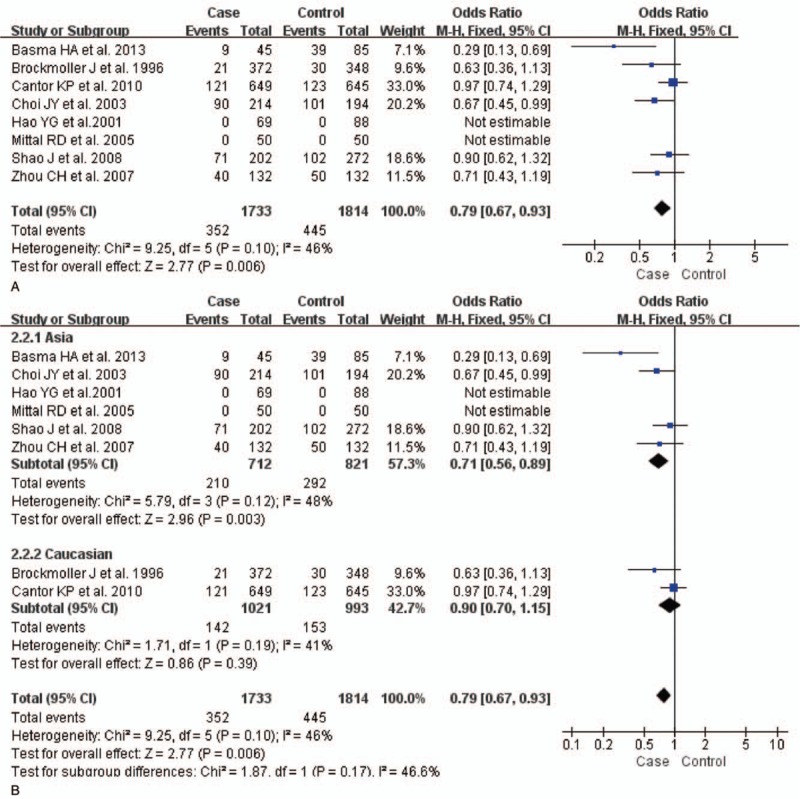
Forest plot showing the meta-analysis outcomes of the dominant model. CI = confidence interval.

**Figure 5:**
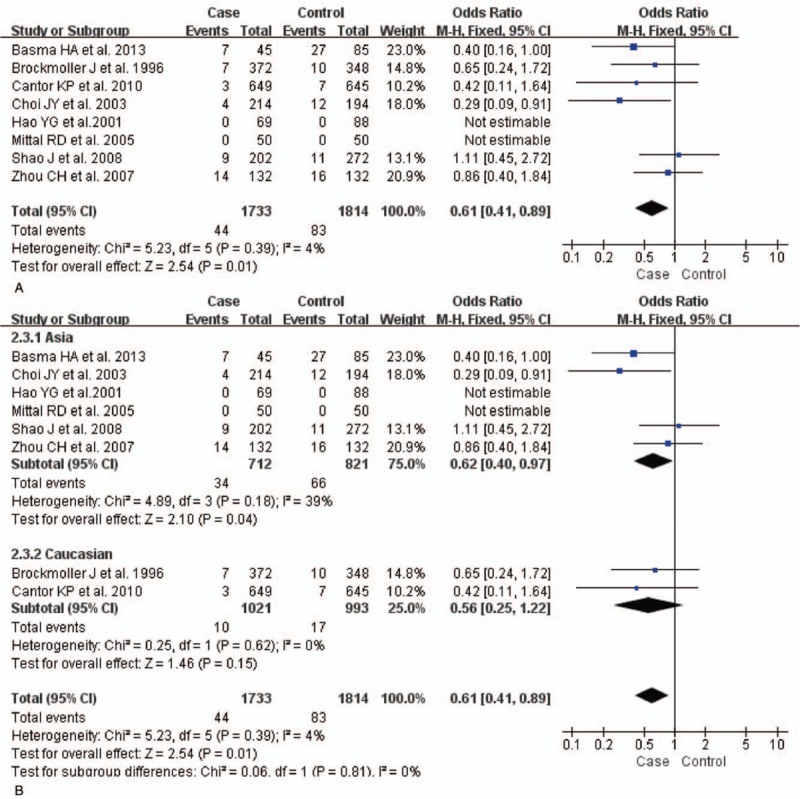
Forest plot showing the meta-analysis outcomes of the recessive model. CI = confidence interval.

**Figure 6:**
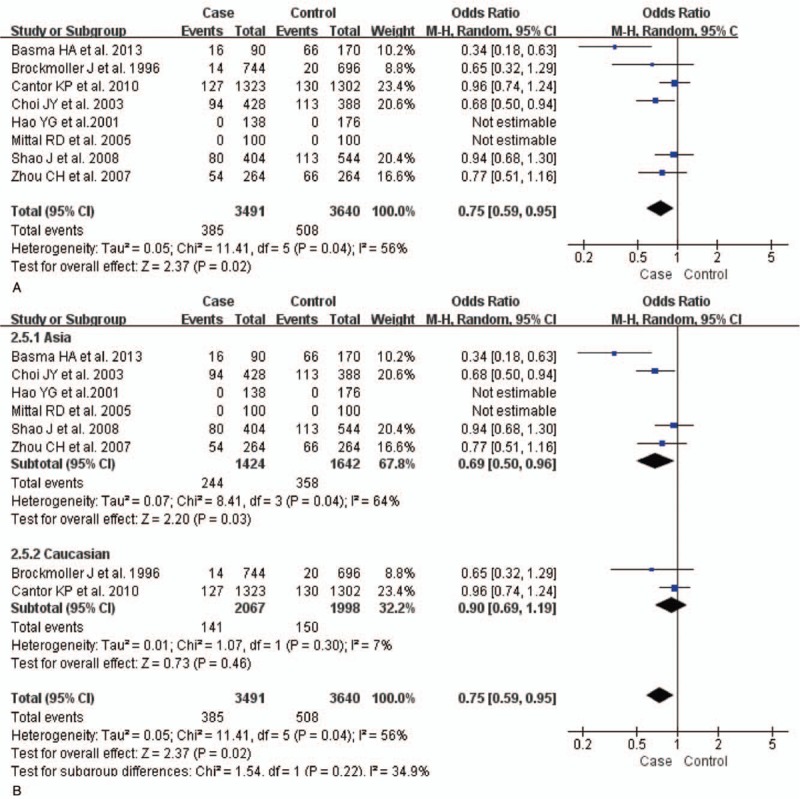
Forest plot showing the meta-analysis outcomes of the allele model. CI = confidence interval.

**Figure 7:**
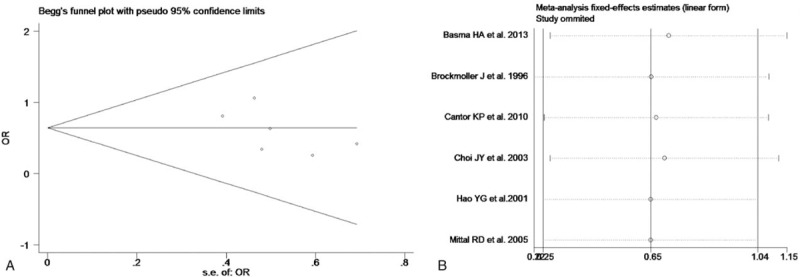
Begg's publication bias and sensitivity analysis plot for the additive model. A, Begg's publication bias. B, Sensitivity analysis. OR = odds ratio.

**Figure 8:**
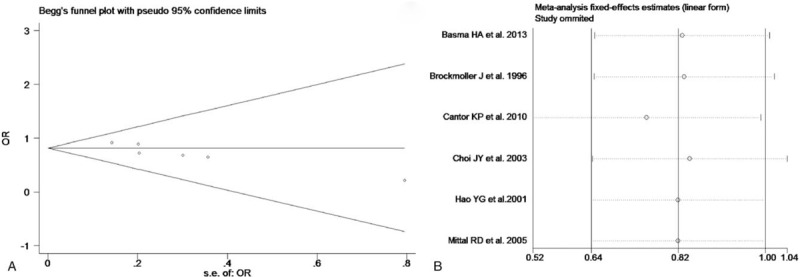
Begg's publication bias and sensitivity analysis plot for the codominant model. A, Begg's publication bias. B, Sensitivity analysis. OR = odds ratio.

**Figure 9:**
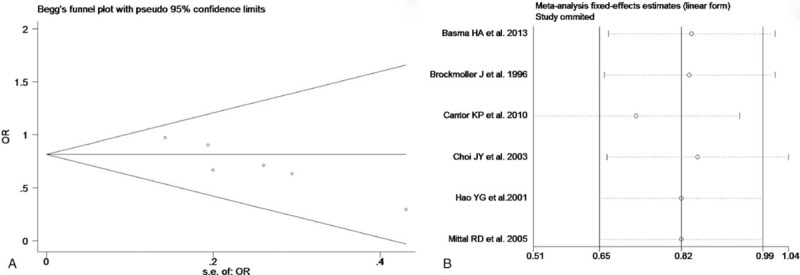
Begg's publication bias and sensitivity analysis plot for the dominant model. A, Begg's publication bias. B, Sensitivity analysis. OR = odds ratio.

**Figure 10:**
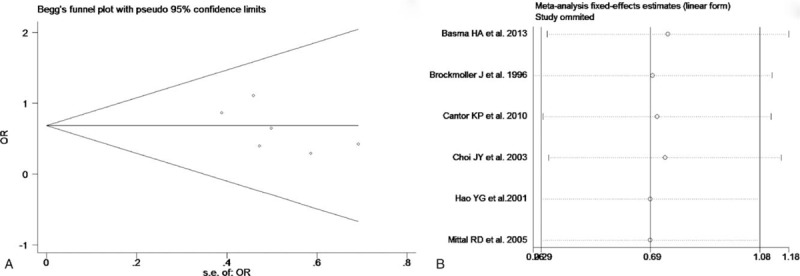
Begg's publication bias and sensitivity analysis plot for the recessive model. A, Begg's publication bias. B, Sensitivity analysis. OR = odds ratio.

**Figure 11:**
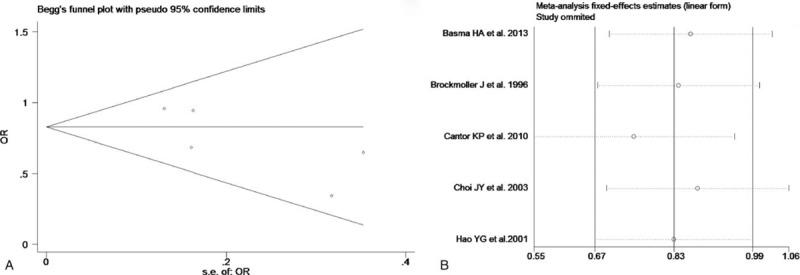
Begg's publication bias and sensitivity analysis plot for the allele model. A, Begg's publication bias. B, Sensitivity analysis. OR = odds ratio.

The test of heterogeneity suggested that data of the allele model were analyzed in a random-effects model, and the recessive, additive, dominant, and codominant models were analyzed in a fixed-effects model. Meta-analysis revealed that there were significant associations between *CYP2E1* gene polymorphisms and bladder cancer in all genetic models (*P* < .05). The combined OR and its 95% CIs were as follows for each model: additive model [OR 0.56; 95% CI (0.38–0.82)] (Fig. 2A); codominant model [OR 0.80, 95% CI (0.67–0.96)] (Fig. 3A); dominant model [OR 0.79; 95% CI (0.67–0.93)] (Fig. 4A); recessive model [OR 0.61; 95% CI (0.41–0.89)] (Fig. 5A); allele model [ORs 0.75; 95% CI (0.59–0.95)] (Fig. 6A). Subgroup study showed that there were also significant associations between *CYP2E1* gene polymorphisms and bladder cancer in Asian people (*P* < .05). However, there were no significant associations between *CYP2E1* gene polymorphisms and bladder cancer in Caucasian individuals (*P* > .05) (Fig. 2B, 3B, 4B, 5B, 6B). Begg funnel plots were largely symmetric (Fig. 7A, 8A, 9A, 10A, 11A), suggesting that there were no publication biases in the meta-analysis. In order to evaluate the stability and reliability of the meta-analysis, we conducted a sensitivity analysis. We sequentially omitted 1 study, and calculated the combined ORs of the remaining studies, but the final conclusions were not changed [there were significant associations between *CYP2E1* gene polymorphisms and bladder cancer in all genetic models (*P* < .05)], which suggest that the results were statistically stable and reliable (Fig. 7B, 8B, 9B, 10B, 11B).

## 4 Discussion

In our study, 8 reports that studied the association of *CYP2E1* gene polymorphisms with bladder cancer risk were analyzed. For the additive, dominant, recessive, codominant, and allele models, 1, 2, 2, 1, and 2 studies, respectively, reported a significant association between *CYP2E1* gene polymorphisms and bladder cancer. Our results revealed that, on the whole, significant associations between *CYP2E1* gene polymorphisms and bladder cancer were found in all genetic models. The subgroup study showed that there are also significant associations between *CYP2E1* gene polymorphisms and bladder cancer in Asian populations. However, no significant associations between *CYP2E1* gene polymorphisms and bladder cancer were identified in Caucasian populations. Thus, these data indicate that *CYP2E1* gene polymorphisms might be a protective factor against bladder cancer in Asian individuals.

The CYP superfamily is an important enzyme and plays a considerable role in the metabolism of endogenous and exogenous compounds, such as numerous carcinogens such as nitrosamines. The enzyme also produce reactive free radicals through oxidation of other compounds, such as ethanol, and may initiate lipid peroxidation and consequently carcinogenesis.^[24,25]^ CYP2E1 represents a major CYP isoform, in the liver, kidney, and other organs and tissues, such as urothelial cells are constitutively expressed this gene.^[13]^ It participates in phase I metabolic oxidization and activates many carcinogenic compounds, such as alkanes, alkenes, and aromatic and halogenated hydrocarbons.^[26,27]^

There is strong experimental evidence that CYP2E1 is an important activator of carcinogenic compounds.^[28,29]^

Several restriction fragment length polymorphisms of the *CYP2E1* have been identified^[30]^ and a variant C2 allele recognized by *RsaI* digestion in the 5′-flanking region of the gene appears to be associated with decreased enzyme activity or noninducibility,^[31]^ some in vitro studies showed that the C2 allele decreased the expression of a reporter gene construct.^[32,33]^ Our results showed that the C2 allele distribution frequency in the control group was significantly higher than that in patients with bladder cancer, suggesting that *CYP2E1* gene polymorphisms might be a protective factor against bladder cancer.

The potential molecular basis for the association between the *CYP2E1* C1/C1 genotype and bladder cancer risk may be related to the expression levels of the gene. The *CYP2E1* C1/C1 genotype shows higher transcriptional activity compared with the *CYP2E1* C2/C2 genotype.^[34]^ Therefore, upon exposure, the enzymatic activity of bladder procarcinogens in C2/C2 subjects may compete poorly compared with C1/C1,^[35–37]^ leading to increased cancer susceptibility. In our subanalysis, there were no significant associations between *CYP2E1* gene polymorphisms and bladder cancer in Caucasian people. This may be because of racial differences in polymorphism distribution.^[38,39]^ Another study found that the frequency of the C2 allele in Asian populations is higher than that in Caucasians.^[40]^ However, this hypothesis requires further investigation before drawing conclusions.

There are some limitations in our study, which need to be taken into consideration when interpreting the results of this meta-analysis. First, the sample size of each study was relatively small, with a total of 1733 patients with bladder cancer and 1814 normal controls investigated in all 8 studies; furthermore, only 2 studies were selected in our subanalysis study. Second, several studies related to the subject were excluded because of a lack of control data. As such, it is hard to make definitive conclusions about the clinical value of *CYP2E1* gene variants and bladder cancer.

In summary, the results of this meta-analysis suggest that the current article adds to the evidence of an association between *CYP2E1* gene polymorphisms and bladder cancer progression. These data suggest that *CYP2E1* gene polymorphisms might be a protective factor against bladder cancer in Asian people. The possible mechanism may occur via *CYP2E1* gene C2 allele mutations, weakening the expression of the gene and enzymatic bioactivation procarcinogens to become ultimate carcinogens and cause genotoxicity. However, studies with larger sample sizes are needed to definitively determine the correlation between *CYP2E1* gene polymorphisms and bladder cancer.

### Uncited References

^[8,9]^.

## Author contributions

**Conceptualization:** Xiangrui Yin, Shengqiang Qian, Yu Guo.

**Data curation:** Xiangrui Yin, Shengqiang Qian, Yu Guo.

**Formal analysis:** Xiangrui Yin, Wei Xiong, Wei Tang, Shengqiang Qian, Yu Guo.

**Funding acquisition:** Xiangrui Yin, Wei Xiong, Wei Tang, Wang Xi, Yu Guo.

**Investigation:** Wei Xiong, Wei Tang.

**Methodology:** Wei Xiong, You Wang, Wei Tang.

**Project administration:** Wei Xiong, You Wang, Wei Tang, Yu Guo.

**Resources:** You Wang, Wei Tang, Wang Xi.

**Software:** You Wang, Wei Tang, Wang Xi, Shengqiang Qian.

**Supervision:** You Wang, Wang Xi, Shengqiang Qian.

**Validation:** You Wang, Wang Xi, Shengqiang Qian.

**Visualization:** You Wang, Wang Xi, Shengqiang Qian.

**Writing – original draft:** Xiangrui Yin, Wang Xi, Yu Guo.

**Writing – review and editing:** Xiangrui Yin.
